# Applying fuzzy logic to assess the biogeographical risk of dengue in South America

**DOI:** 10.1186/s13071-019-3691-5

**Published:** 2019-09-05

**Authors:** David Romero, Jesús Olivero, Raimundo Real, José Carlos Guerrero

**Affiliations:** 1 Laboratorio de Desarrollo Sustentable y Gestión Ambiental del Territorio (LDSGAT), Instituto de Ecología y Ciencias Ambientales (IECA), Facultad de Ciencias, Universidad de la República, Iguá 4225, 11400 Montevideo, Uruguay; 2 Departamento de Biología Animal, Grupo de Biogeografía, Diversidad y Conservación, Facultad de Ciencias, Universidad de Málaga, Bulevar Louis Pasteur, 31, 29010 Málaga, Spain

**Keywords:** *Aedes aegypti*, *Ae. albopictus*, Favorability function, Fuzzy operators, Vector-illness interaction

## Abstract

**Background:**

Over the last decade, reports about dengue cases have increase worldwide, which is particularly worrisome in South America due to the historic record of dengue outbreaks from the seventeenth century until the first half of the twentieth century. Dengue is a viral disease that involves insect vectors, namely *Aedes aegypti* and *Ae. albopictus*, which implies that, to prevent and combat outbreaks, it is necessary to understand the set of ecological and biogeographical factors affecting both the vector species and the virus.

**Methods:**

We contribute with a methodology based on fuzzy logic that is helpful to disentangle the main factors that determine favorable environmental conditions for vectors and diseases. Using favorability functions as fuzzy logic modelling technique and the fuzzy intersection, union and inclusion as fuzzy operators, we were able to specify the territories at biogeographical risk of dengue outbreaks in South America.

**Results:**

Our results indicate that the distribution of *Ae. aegypti* mostly encompasses the biogeographical framework of dengue in South America, which suggests that this species is the principal vector responsible for the geographical extent of dengue cases in the continent. Nevertheless, the intersection between the favorability for dengue cases and the union of the favorability for any of the vector species provided a comprehensive map of the biogeographical risk for dengue.

**Conclusions:**

Fuzzy logic is an appropriate conceptual and operational tool to tackle the nuances of the vector-illness biogeographical interaction. The application of fuzzy logic may be useful in decision-making by the public health authorities to prevent, control and mitigate vector-borne diseases.

## Background

Dengue is one of the diseases with most epidemiological global relevance in the last decades [1–9]. Over this century, dengue has become a growing public health problem and about half of the world’s population is currently at risk of dengue infection [7, 8, 10–12]. This is especially a concern in South America, where historical records of dengue epidemic outbreaks report upsurges every three or five years from the seventeenth century until the first half of the twentieth century [13, 14].

*Aedes* mosquitoes, namely the yellow fever mosquito (*Aedes aegypti*) and the Asian tiger mosquito (*Ae. albopictus*), are the most important dengue vectors in the world [15–17]. The number of studies about the mosquitoes of the genus *Aedes* as transmission vectors of human infectious diseases has recently increased remarkably [7, 10, 18–21]. Different authors have studied relevant aspects of mosquito-dengue relationships from phylogenetic [22], ecological [17–19], physicochemical [20, 23], genetic [21] and biogeographical perspectives [7, 10, 24, 25].

A biogeographical approach to the study of zoonotic diseases, known as pathogeography, has contributed with relevant advances in the knowledge of infectious disease macroecology and distribution [26–29]. It has also provided a proper analytical framework for the study of vector-illness interaction useful for management or surveillance. Species distribution models (hereinafter SDM) have been particularly used to investigate the environmental drivers for the distribution of *Aedes* species [25], to map the global distribution of the *Aedes* species according to the effect of temperature [10, 25], precipitation, and some land cover variables [7, 10], or to forecast the possible effects of climate change scenarios for *Aedes* species distributions [10]. Other studies also took into account economic information [4], or focused on predicting and determining the global burden of dengue [4, 24]. However, the biogeographical framework of vector-illness interaction that could reveal the large-scale risk of dengue occurrence remains poorly understood.

The current range occupied by *Aedes* mosquitoes (*Ae. aegypti* and *Ae. albopictus*) in South America is wider than the known dengue cases. For some reason not yet fully elucidated, there are territories occupied by *Aedes* vectors with and without dengue cases. This suggests that the relationship between the occurrence of *Aedes* mosquito populations and cases of dengue is not clear-cut, and that a fuzzy-logic approach is worth considering. In contrast to crisp logic, Zadeh [30] proposed the fuzzy set theory, which avoids the use of discrete true-or-false syllogisms, thus conferring a conceptual malleability suitable for real-life situations. Salski and Kandzia [31] emphasized the continuous character of nature, which implies that living beings are distributed in time and space essentially in a gradual and fuzzy manner. A fuzzy logic approach is consequently useful for processing and modelling environmental data [32]. Thus, the application of fuzzy logic could be helpful to recognize the biogeographical vector-illness interaction and the dynamism in the risk of dengue occurrence, and to establish the biogeographical framework in which the disease occurs.

Fuzzy logic led to the notion of environmental favorability, a concept related to, but different from, probability of occurrence [33]. Favorability functions can be used in SDM, and are particularly helpful when models of several species are involved in the study, as they allow the comparison between models for species or cases differing in prevalence, using fuzzy logic tools [28, 34–38].

In this study, our aims were to establish the biogeographical context in which dengue cases occur in South America and to map the areas favourable for new cases to occur. We assessed vector-illness biogeographical relationship using fuzzy logic to determine the different environmental drivers that favor the occurrence of both *Aedes* vectors and of dengue cases. We aimed to identify the territories more at biogeographical risk of dengue outbreaks, which may be helpful in order to apply measures for the management and control of this recurrent disease.

## Methods

### Study area and species range

**Fig. 1:**
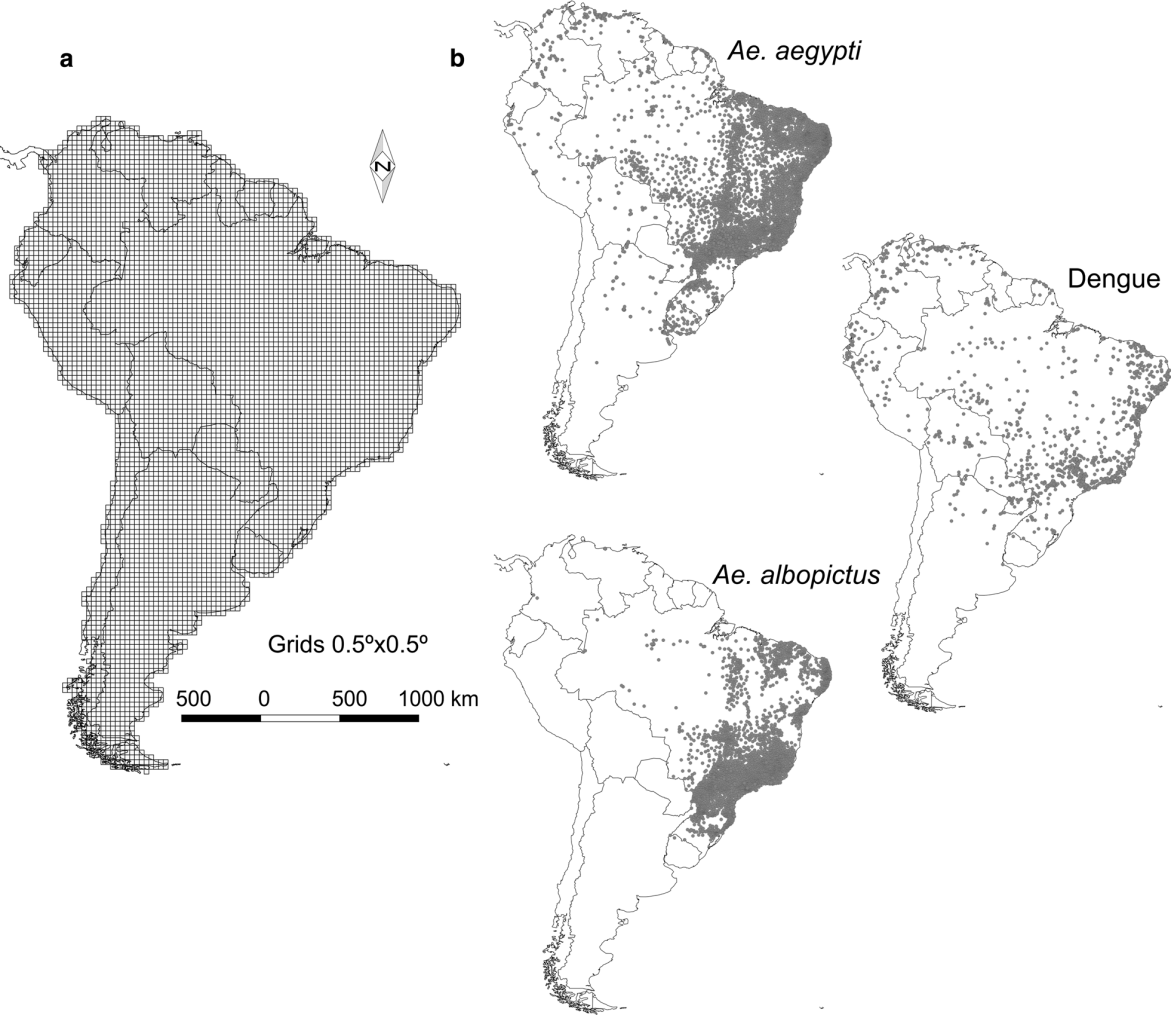
Study area and distribution data: **a** the grid of 0.5° latitude × 0.5° longitude squares in which the study area was divided to represent the occurrence data; **b** occurrence data of vectors and dengue infection cases. The grid layer was created with the tool “Create grid” of the software QGIS (www.qgis.org). The country layer was obtained from https://www.naturalearthdata.com and licensed CC BY. The maps were developed using QGIS in the composer tool. The final composition was created using CorelDRAW X8

We analyzed *Ae. aegypti*, *Ae. albopictus* and dengue virus occurrences on a 0.5° × 0.5° grid (6430 cells of approximately 50 km × 50 km at the equator) to identify the biogeographical relationship between *Aedes* vectors and dengue cases. We used grids instead of geographical locations, thus solving a large part of the spatial autocorrelation problems derived from sampling bias or observation spatial clustering. Dengue virus occurrences were obtained from global occurrence records published from 1960 to 2012 in Messina et al. [5], with 731 grid cells with confirmed cases, which cover 11.37% of South America (Fig. 1). *Aedes aegypti* and *Ae. albopictus* occurrences were obtained from the global compendium of *Aedes aegypti* and *Ae. albopictus* occurrence [8] and from the Faculty of Science of the Republic University of Uruguay (inbuy.fcien.edu.uy, accessed in May 2017), with data spanning from 1960 to 2013, and from 1986 to 2014, respectively. *Aedes aegypti* was confirmed to occur in 1688 cells whereas *Ae. albopictus* presence was confirmed in 957 cells, covering 26.25 and 14.88% of South America, respectively (Fig. 1).

### Environment predictors and distribution modelling

We modelled the distribution of the two vector species and of dengue virus occurrence (the target variables) on the basis of a set of explanatory variables that could potentially affect them at the spatial resolution here applied [39, 40] (Table 1). The explanatory variables were related to different environmental factors that could determine the area occupied by both *Aedes* species and the extent of dengue virus occurrence in South America: spatial configuration, topography, climate (rainfall and temperature), hydrology, land use and other human activities (Table 1).

**Table 1:** Explanatory variables used in *Ae. aegypti*, *Ae. albopictus* and dengue virus models in South America. Climate variables which do not have a pairwise correlation value above 0.80 according to Spearmanʼs test are shown in bolditalic

Abbreviation	Variable	Abbreviation	Variable
SP		Spatial lineal combination (*ysp*)^a^	
Topography			
A	Mean altitude (m)^b^	S	Slope (◦) (calculated from altitude)
D_A_	Difference altitude (m) (calculated from altitude)	O_N/S_	Orientation N/S (calculated from slope)
Climatic variables			
***BIO***_**1**_	***Mean annual temperature (°C)*** ^c^	BIO_11_	Mean annual temperatures of the coldest quarter (°C)^c^
BIO_2_	Mean diurnal range temperatures (°C)^c^	***BIO***_**12**_	***Annual precipitation (mm)*** ^c^
BIO_3_	Isotermality (BIO_2_/BIO_17_)(*100) (°C)^c^	BIO_13_	Precipitation of the wettest month (mm)^c^
BIO_4_	Seasonal temperatures (°C)^c^	BIO_14_	Precipitation of the driest month (mm)^c^
BIO_5_	Maximum temperatures of the warmest month (°C)^c^	***BIO***_**15**_	***Seasonal precipitation (coeficiente de variación) (mm)*** ^c^
BIO_6_	Minimum temperatures of the coldest month (°C)^c^	BIO_16_	Precipitation of wettest quarter (mm)^c^
***BIO***_**7**_	***Annual temperatures range (BIO***_**5**_–***BIO***_**6**_)^c^	BIO_17_	Precipitation of dry quarter^c^
BIO_8_	Mean annual temperatures of the wetter quarter^c^	***BIO***_**18**_	***Precipitation of warmest quarter*** ^c^
BIO_9_	Mean annual temperatures of the dry quarter^c^	***BIO***_**19**_	***Precipitation of coldest quarter*** ^c^
BIO_10_	Mean annual temperatures of the warmest quarter^c^		
Hydrology			
DistRiver	Minimum distance to rivers (km)^d^	SumRiver	Sum of km of rivers per grid (km)^d^
Land use			
Forests	Forests (%)^e^	Crops	Crops (%)^e^
NatField	Natural field (%)^e^	BareSoil	Bare soil (%)^e^
FlooVeg	Flooding vegetation (%)^e^		
Human activities			
PopDen	Population density^f^	DistRoad	Minimum distance to paved roads (km)^h^
DistUrban	Minimum distance to urban centers (km)^g^		

^a^Spatial variables, latitude and longitude, were generated using QGIS (www.qgis.org) according to the vector geometry tools: (i) with “centroids of polygons” the centroid of each grid was calculated, and (ii) with “Export/Add columns of geometry” values of length and latitude expressed in the 1984 World Geodetic System were assigned to each centroid (WGS84). The spatial variable used in the multivariate modelling procedure is the linear polynomial combination (ysp) resulting from a spatial logistic regression

^b^United States Geological Survey. GTOPO30. Land Processes Distributed Active Archive Center. EROS Data Center, https://www.usgs.gov/centers/eros/science/usgs-eros-archive-digital-elevation-global-30-arc-second-elevation-gtopo30?qt-science_center_objects=0#qt-science_center_objects. 1996 (Accessed April 2016)

^c^WorldClim—Global Climate Data available. Described in: Fick, S. E. and R. J. Hijmans. Worldclim 2: New 1-km spatial resolution climate surfaces for global land areas. International Journal of Climatology. 2017. In: http://www.worldclim.org/ (Accessed May 2016)

^d^United States Geological Survey. HydroShed. Hydrological data and maps based on SHuttle Elevation Derivatives at multiple Scales. Available in: http://hydrosheds.cr.usgs.gov/index.php/ (Accessed May 2016)

^e^GlobCover 2009. Global land cover map. 2006. Avalaible at: http://due.esrin.esa.int/page_globcover.php (Accessed April 2016)

^f^Gridded Population of the World (GPW), v4. Socioeconomic Data and Applications Center (SEDAC). A Data Center in NASA’s Earth Observing System Data and Information System (EOSDIS). Hosted by CIESIN at the Columbia University. 2010. (Accessed June 2016)

^g^Natural Earth Data. North American Cartographic Information Society (NACIS). Available at: http://www.naturalearthdata.com/ (Accessed April 2016)

^h^Diva-Gis 1.4, Plant Genetic Resources Newsletter. Available in: http://www.diva-gis.org/ (Accesed April 2016)

To define the spatial structure of each distribution, we considered a polynomial trend-surface analysis [41] that included a quadratic and cube effect of latitude and longitude and interactions between them. Spatial structure is known to be functional in biogeography, as purely spatial trends derive from biological processes such as history, spatial ecology and population dynamics [42]. Spatial structure is known to be functional in biogeography, as purely spatial trends derive from biological processes such as history, spatial ecology and population dynamics [42]. To define the spatial structure of each distribution, we considered a polynomial trend surface analysis [41] that included a quadratic and cube effect of latitude and longitude and interactions between them. For this, we performed a logistic regression of *Ae. aegypti*, *Ae. albopictus* and dengue virus distribution data on latitude (Lat), longitude (Lon), Lat2, Lon2, Lat3, Lon3, Lat × Lon, Lat2 × Lon and Lat × Lon2. Specifically, we performed a backward stepwise logistic regression with each event (both *Aedes* vectors and dengue cases) and those nine spatial terms as predictor variables in order to remove the non-significant spatial terms from models [41]. In this way, in the modelling procedure we included the resulting lineal combinations (*ysp*) as the spatial variable without non-significant spatial terms.

We used this spatial lineal combination (*ysp*) and the rest of variables listed in Table 1 (environmental factors) to produce distribution models according to all the explanatory factors together. To do this, we first analysed the effect of each explanatory variable on each target variable on a bivariate basis, by performing a logistic regression of each target variable on each explanatory variable separately. So, as Miller et al. [43] indicated, including the variation of the response variables separated into environmental and spatial components (represented by a trend surface of geographical coordinates) is a way to quantify the spatial dependence in distribution models ([44, 45], among others). We controlled the false discovery rate (FDR) with the aim of avoiding the increase in type I errors arising from the number of variables used in the analyses [46]. An explanatory variable was selected only when it was significantly related to the target variable (*P* < 0.05) under a FDR of *q* < 0.05, with *q* being the false discovery rate. Then, we calculated Spearman correlation coefficients to control multicollinearity between the selected explanatory variables. Out of any group of explanatory variables whose pairwise correlation value was higher than 0.80, we retained the variable most significantly related with the distribution of the target variable. In this way we obtained a filtered set of potentially explanatory variables for each target variable.

Finally, we performed a forward-backward stepwise logistic regression of the target variable on the polynomial combination of the spatial structure (*ysp*) and the filtered set of environmental variables, which produced increasingly more complex multivariate models while avoiding the inclusion of redundant variables. We used Akaike’s information criterion (AIC) to select the multivariate model that best-balanced information and parsimony (AIC; [47]). All analyses mentioned so far were performed with the *fuzzySim* R package [38]. Then, we evaluated the relative weight of each variable included in the models through the Wald parameter [48] using the survey package [49, 50]. Variables with non-significant coefficients left in the model (Chi-square test, *P* < 0.05) were eliminated until we obtained a model with all the coefficients significantly different from zero according to Crawley’s [51] procedure.

Then we used the Favorability Function according to Real et al. [33] and Acevedo and Real [52].

$${\text{F}} = {{\left[ {{\text{P}}/\left( {1 - {\text{P}}} \right)} \right]} \mathord{\left/ {\vphantom {{\left[ {{\text{P}}/\left( {1 - {\text{P}}} \right)} \right]} {\left[ {\left( {{\text{n}}1/{\text{n}}0} \right) + \left( {{{\text{P}} \mathord{\left/ {\vphantom {{\text{P}} {\left[ {1 - {\text{P}}} \right]}}} \right. \kern-0pt} {\left[ {1 - {\text{P}}} \right]}}} \right)} \right]}}} \right. \kern-0pt} {\left[ {\left( {{\text{n}}1/{\text{n}}0} \right) + \left( {{{\text{P}} \mathord{\left/ {\vphantom {{\text{P}} {\left[ {1 - {\text{P}}} \right]}}} \right. \kern-0pt} {\left[ {1 - {\text{P}}} \right]}}} \right)} \right]}}$$

where F is the environmental favorability (ranging between 0 and 1), P is the probability of occurrence obtained from the multivariate logistic regression performed for each target variable, n1 is the number of presences and n0 in the number of absences, in each case. This analysis was carried out with the *fuzzySim* R package [38].

Favorability values factor out the weight of the initial species presence/absences ratio, inherent to any probability function [33, 52] and, thus, depend exclusively on the effect of the environmental conditions of the territory on the distribution under analysis. In addition, local favorability reflects the degree of membership of the locality in the fuzzy set of areas favorable for the occurrence of the event, so allowing the comparison between models through fuzzy logic tools [36, 52].

In this way, we obtained favorability models for the occurrence of the two *Aedes* vectors (*Ae. aegypti* and *Ae. albopictus*) and of dengue in South America, F-*Ae*. *aegypti*, F-*Ae. albopictus* and F-dengue, respectively. We evaluated the discrimination and classification capacity of the models with the *modEva* R package [53]. The discrimination ability of the models was evaluated using the area under the curve (AUC) [54], and the classification capacity was estimated through the model sensitivity, specificity, kappa and correct classification rate (CCR), using the value of F = 0.5 as classification threshold. We checked the autocorrelation spatial using the Moran’s I spatial autocorrelation statistic from the residuals of the models [55].

According to the thresholds proposed by Muñoz and Real [56], we calculated the number of grid cells in each South American country classified as highly favorable (F ≥ 0.8), for which the favorability odds are more than 4:1 in favor, hereinafter at high risk, and of intermediate favorability (0.2 < F < 0.8), which odds are under 4:1 and above 1:4 in favor, hereinafter vulnerable, for *Aedes* vectors, for dengue cases, and for vector-dengue cases simultaneously (see below).

### Biogeographical vector-dengue relationships and dengue risk maps

We used the fuzzy modelling approach to assess the vector-dengue biogeographical interaction in South America. The logic underlying fuzzy sets was applied to the favorability function to indicate to what degree each grid cell belongs to the set of favorable areas for the presence of each species [52]. Then we used fuzzy logic tools to analyze the fuzzy vector-dengue biogeographical interactions and to detect the territories at high risk or vulnerable to new dengue cases.

Based on the values of F-*Ae. aegypti*, F-*Ae. albopictus* and F-dengue models, we calculated the fuzzy intersection (minimum favorability value for two events at a given location) [30] to identify the fuzzy set of areas simultaneously favorable for dengue outbreaks and for any of the two species separately (i.e. F-*Ae. aegypti* ∩ F-dengue and F-*Ae. albopictus* ∩ F-dengue). Then, we analyzed how the favorability for each vector presence and for the occurrence of dengue cases changed along the gradient of favorability intersection (i.e. of shared favorability for both vector and disease). To this aim, we established 10 bins of equal-range F-*Ae. aegypti* ∩ F-dengue values and F-*Ae. albopictus* ∩ F-dengue values and calculated in each bin the average favorability values for the corresponding vector species and for dengue virus. If the vector species is a limiting factor in the distribution of the disease, then the favorability for dengue should be equal to or lower than that for the mosquito along the shared favorability range.

We also calculated to what extent the favorable areas for dengue (F-dengue) are contained in those for F-*Ae. aegypti* and for F-*Ae. albopictus* models, by applying the fuzzy inclusion equation [57]:

$$I\left( {A,B} \right) = \frac{{\left| {A \cap B} \right|}}{\left| A \right|}$$

which indicates how much the set A is included in the set B. In this way, we calculated the inclusion of one into the other for the models F-*Ae. aegypti*, F-*Ae. albopictus* and F-dengue, and also for vector-dengue intersections (i.e. F-*Ae. aegypti* ∩ F-dengue and F-*Ae. albopictus* ∩ F-dengue). Those fuzzy inclusion operations are defined in terms of the cardinal of each fuzzy set (i.e. the sum of the favorabilities values of all the grids). Thus, for example, the cardinal of F-*Ae. aegypti* ∩ F-dengue divided by the cardinal of F-dengue indicates the degree of inclusion of the distribution of dengue into that of *Aedes aegypti*.

To obtain the comprehensive biogeographical risk map for dengue in South America in the current context of vector-dengue biogeographical relationship, we identified the fuzzy set of areas simultaneously favorable for dengue outbreaks and for any of the two vector species. To do this we first calculated the fuzzy union of the favorability for any vector species, F-*Ae. aegypti* ∪ F-*Ae. albopictus* (or maximum favorability value for any of them), which can identify the fuzzy set of areas favorable to either vector species [30]. Then, we calculated the fuzzy intersection between F-*Ae. aegypti* ∪ F-*Ae. albopictus* and F-dengue [(F-*Ae. aegypti* ∪ F-*Ae. albopictus*) ∩ F-dengue].

## Results

### Favorable conditions for vectors and dengue cases

The variables that were significantly associated with the occurrence of each vector species and with dengue cases are shown in Table 2 (Additional file 1: Table S1). All the factors explained to some extent the occurrence of both vectors and dengue, with the exception of the hydrology for *Ae. albopictus* and hydrology and land use for dengue.

**Table 2:** Predictor variables included in *Ae. aegypti*, *Ae. albopictus* and dengue cases favorability models. Signs in brackets show the positive or negative relationship between favorability and the variables in the models. The Wald parameter indicates the relative weight of every variable in each model. Variable abbreviations are given in Table 1

Environmental factor	*Ae. aegypti*	Wald	*Ae. albopictus*	Wald	Dengue cases	Wald
Spatial situation	Sp	1221.597	Sp	770.6308	Sp	252.8375
Topography			A (−)	15.03676	A (+)	30.7381
	O _N/S_ (+)	21.09944	O_N/S_ (+)	65.61643	O_N/S_ (+)	13.52308
	S (+)	11.75523	S (+)	8.108446		
Climatic	BIO_1_ (+)	73.15295	BIO_1_ (+)	24.7797	BIO_1_ (+)	83.51969
	BIO_7_ (+)	7.520641	BIO_7_ (+)	18.91607		
	BIO_12_ (+)	8.464809	BIO_12_ (+)	32.34416		
					BIO_15_ (−)	11.84533
	BIO_19_ (−)	17.86841			BIO_19_ (−)	25.6279
Hydrology	SumRiver (−)	10.33488				
Land use	Crops (+)	19.87312	Crops (−)	6.809295		
			NatFields (−)	6.809295		
Human activities	PopDen (+)	21.1916	PopDen (+)	7.74626	PopDen (+)	33.15417
	DistUrban (−)	148.1178	DistUrban (−)	43.05502	DistUrban (−)	173.8293
			DistRoad (−)	7.287445		

The distribution of dengue was favored in territories of a certain elevation (435.06 m.a.s.l. on average), of predominant orientation towards the south, of high mean annual temperatures (23.13 °C on average), with low precipitation in the colder months (185.86 mm on average), few differences between maximum and minimum precipitations, high population density (221 inhabitants/km^2^ on average) and moderate distance to urban centers (7750 m on average).

The distribution of both *Aedes* vectors was favored by similar variables with similar effect (positive or negative), except for the land use factor. A high proportion of crops was favorable for *Ae. aegypti* while it was unfavorable for *Ae. albopictus*. According to the Wald test, in both *Ae. aegypti* and dengue models, the three most explanatory variables were the spatial structure, closeness to urban centers and mean annual temperature (Table 2). The spatial structure, North-South orientation and proximity to urban centers were the three most explanatory variables for *Ae. albopictus*.

**Fig. 2:**
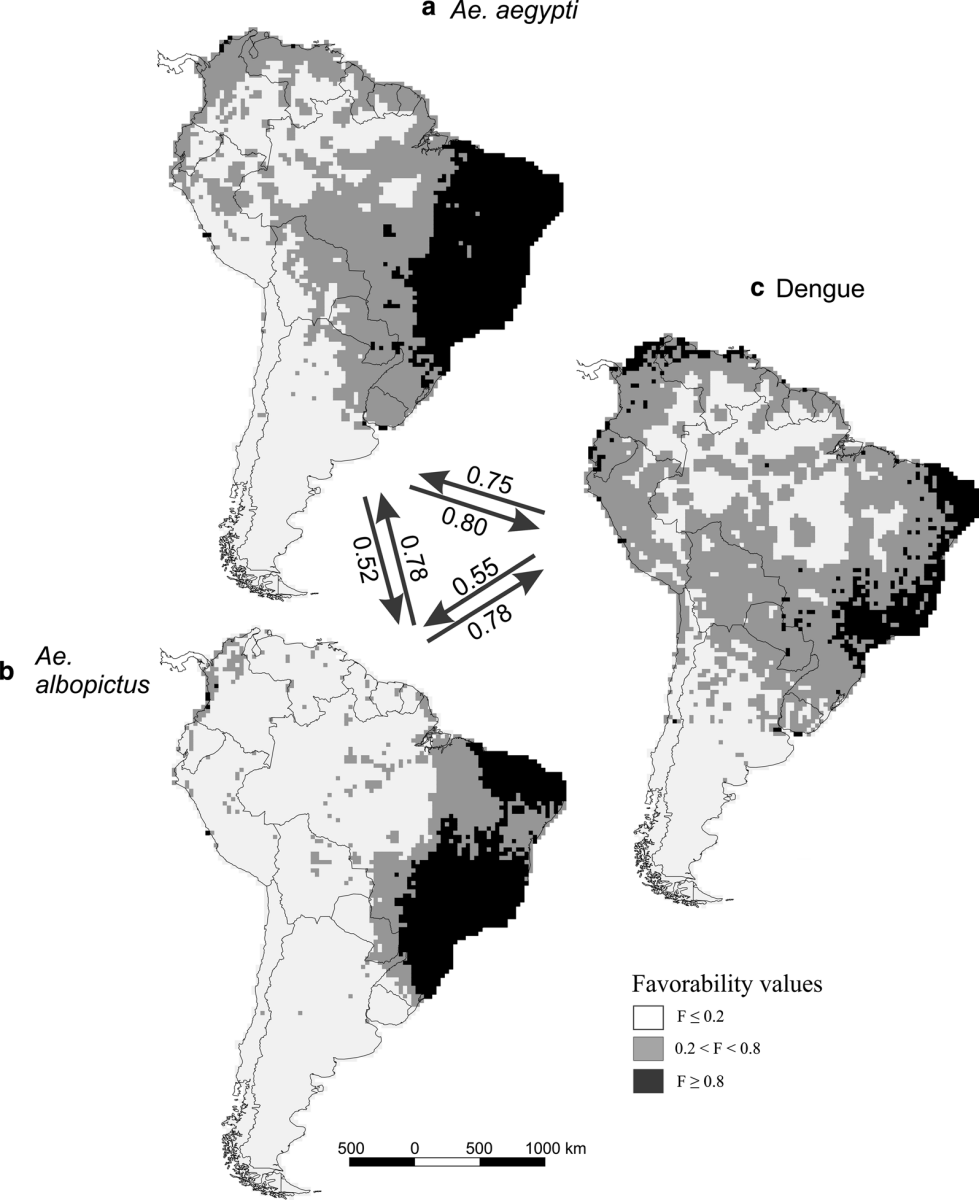
Favorability models of: **a** *Aedes aegypti*, **b** *Ae. albopictus* and **c** dengue cases. Favorable areas are shown in black (favorability values or F ≥ 0.8), grey (0.2 < F < 0.8) and white (F ≤ 0.2). The arrows show inclusion values between the different models, one into the other. The maps were developed using QGIS (www.qgis.org) in the composer tool. The final composition was created using CorelDRAW X8

In Fig. 2 we show the cartographic favorability models for the vector species and for dengue (F-*Ae. aegypti*, F-*Ae. albopictus* and F-dengue) separately, with values grouped in three favorability classes: F values lower than 0.2 indicate low favorability, values between 0.2 and 0.8 indicate vulnerable areas, and values higher than 0.8 indicate areas at high risk [58]. The F-*Ae. aegypti* model depicted a large principal nucleus of high risk in Brazil, and some dispersed high-risk cells in Venezuela, Colombia, Peru, Paraguay, Argentina and Uruguay. The F-*Ae. albopictus* map revealed a main high-risk nucleus in Brazil, and some dispersed high-risk cells in Colombia and Peru. The F-dengue model detected two main nuclei of high risk for the occurrence of dengue cases, one in Brazil and another, more dispersed, in Venezuela, Colombia and Ecuador. Some dispersed high-risk cells are also found in Peru, Guyana, Surinam, Paraguay and Uruguay. The F-dengue model detected at least one vulnerable grid cell (0.2 < F < 0.8) in every South American country. Although only 11.37% (731 squares) of the total analyzed squares (*n* = 6430) have recorded dengue cases, 60.14% (3867) of the squares showed at least vulnerable conditions (F > 0.2) according the F-dengue model, while 8.94% of the cells (575) were at high risk (F ≥ 0.8).

Both *Aedes* and dengue favorability models attained general acceptable scores according to the parameters considered to assess discrimination and classification capacities (Table 3). Discrimination (AUC) was always higher than 0.86, which is “excellent” according to Hosmer and Lemeshow [59]. Sensitivity values were always higher than 0.79, specificity was always higher than 0.74 and CCR was higher than 0.75. Kappa was higher than 0.6 for both *Aedes* vectors, which is “substantial” according to Landis and Kock [60]; it was 0.31, which is “fair”, for dengue cases. On the other hand, according to the analysis of residuals, we detected a minor autocorrelation (Moran’s I < 0.019), or approximately zero, below 1600 km and only in the *Ae. albopictus* model. These results indicate that there is no relevant spatial autocorrelation resulting from sampling bias with the grid system employed [55]. None of the Moran’s I-values were significant in the *Ae. aegypti* or the dengue models. The residuals did not show problems of spatial autocorrelation in our models and therefore we did not find relevant effects of spatial autocorrelation that invalidate our results.

**Table 3:** Comparative assessment of models for *Aedes aegypti*, *Ae. albopictus* and dengue cases, as well as the fuzzy intersection between the vector species and dengue cases, according to their discrimination and classification capacity

Evaluation indices		Favorability models			Vector-dengue favorability intersection	
		*Ae. aegypti*	*Ae. albopictus*	Dengue cases	*Ae. aegypti* *∩* dengue	*Ae. albopictus* *∩* dengue
Discrimination						
AUC		0.914	0.966	0.862	0.844	0.794
Classification						
Sensitivity		0.791	0.918	0.819	0.640	0.511
Specificity		0.871	0.901	0.742	0.848	0.881
CCR		0.850	0.903	0.751	0.824	0.839
Kappa		0.630	0.682	0.312	0.358	0.329

*Abbreviations*: AUC, area under the ROC (receiving operating characteristic) curve; CCR, correct classification rate

### Vector-dengue biogeographical interactions

Compared to the F-dengue model, both vector-disease intersections improved classification capacity according to kappa, CCR and specificity, whereas sensitivity and discrimination capacity decreased (Table 3).

**Fig. 3:**
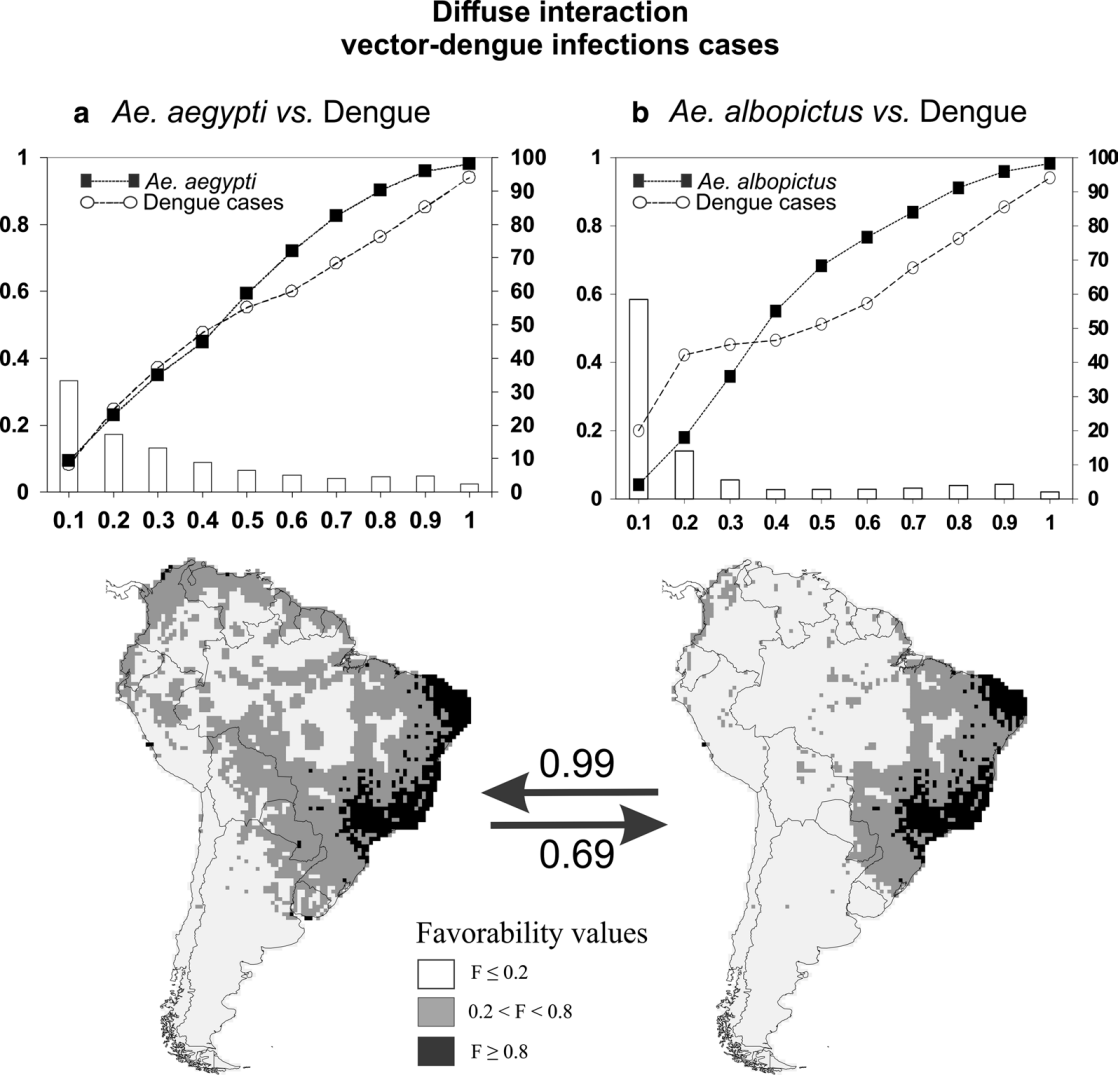
Plots and maps show the fuzzy intersection (simultaneous favorability) between the favorability for: **a** *Ae. aegypti* and dengue infection cases; and **b** favorability for *Ae. albopictus* and dengue infection cases. Fuzzy intersection values are shown on the horizontal axes (ranging from 0.1 to 1), grouped in 10 bins of values of equal favorability range. The average favorability values for both mosquito vectors in each bin are represented by solid lines and filled squares, and for dengue infection cases by dashed lines and blank circles, (on the left vertical axes ranging from 0 to 1). Columns represent the percentage of grid cells at each fuzzy intersection bin (on the right vertical axes). On maps, the arrows show inclusion values between both fuzzy intersection models (ranging from 0 to 1). The graphics were made using LibreOffice (https://es.libreoffice.org). The maps were developed using QGIS (www.qgis.org) in the composer tool. The final composition was created using CorelDRAW X8

In Fig. 3 we show the fuzzy intersection between the favorability for dengue and vector species for both *Ae. aegypti* and *Ae. albopictus* in South America. *Aedes aegypti* and dengue favorability values increased together until a fuzzy intersection of 0.5 was reached; then, both continued to increase with higher favorability values for the mosquito (Fig. 3a). The intersection between *Ae. albopictus* and dengue favorability values indicated that dengue cases had higher favorability values than the mosquito up to fuzzy intersection = 0.3; after that point, the vector showed higher favorability values than the disease.

According to the intersection between F-*Ae. aegypti* and F-dengue (Table 4), nine of the 14 South American countries have more than 50% of the country surface area at least vulnerable (F > 0.2) to dengue-cases occurrence transmitted by *Ae. aegypti*. In contrast, only Brazil has more than 50% of the country at least vulnerable (F > 0.2) to dengue-cases occurrence transmitted by *Ae. albopictus*. Seven South American countries have some locations at high risk (F ≥ 0.8) based on the intersection between F-*Ae. aegypti* and F-dengue. Two countries, Brazil and Peru, have locations at high risk of dengue occurrence due to *Ae. albopictus*, based on the intersection between the F-*Ae. albopictus* and F-dengue models (Table 4).

**Table 4:** Percentages of the country surface with intermediate and high risk (F > 0.2, and F ≥ 0.8, respectively) of both vectors (*Aedes aegypti* and *Ae. albopictus*), of dengue cases, and of vector-dengue favorability intersection (with respect to the total number of grid cells per country in the leftmost column). Countries were ordered from highest to lowest percentage of the country surface of dengue cases detected in Messina et al. [5]

Country	Cells by country	% of risk for *Ae. aegypti*		% of risk for *Ae. albopictus*		% of risk for dengue cases		% of risk intersection F-*Ae*. *aegypti-F-*dengue		% of risk intersection F-*Ae*. *albopictus-*F-dengue		% of the country with dengue cases
		F > 0.2	F ≥ 0.8	F > 0.2	F ≥ 0.8	F > 0.2	F ≥ 0.8	F > 0.2	F ≥ 0.8	F > 0.2	F ≥ 0.8	
Brazil	2860	84.056	42.343	58.776	33.741	69.720	16.049	67.552	15.839	53.497	14.196	16.958
Colombia	349	48.424	1.146	19.198	1.146	70.201	11.461	57.593	1.146	21.490	0.000	13.181
Venezuela	365	60.274	0.274	4.932	0.000	64.932	11.781	47.397	0.274	2.740	0.000	11.507
Peru	509	29.666	0.393	4.322	0.196	79.371	0.786	25.344	0.393	4.322	0.196	8.251
Bolivia	325	60.000	0.000	5.538	0.000	91.385	0.000	76.615	0.000	8.000	0.000	11.692
Paraguay	182	89.560	4.396	40.659	2.747	97.253	3.846	87.363	1.099	40.659	0.000	19.780
Argentina	1062	13.653	0.094	0.659	0.094	19.115	0.188	10.829	0.094	0.659	0.000	1.507
Ecuador	82	46.341	0.000	12.195	0.000	96.341	15.854	46.341	0.000	12.195	0.000	18.293
French Guyana	32	78.125	0.000	15.625	0.000	62.500	0.000	53.125	0.000	9.375	0.000	12.500
Guyana	72	58.333	0.000	4.167	0.000	58.333	1.389	61.111	0.000	5.556	0.000	5.556
Surinam	65	73.846	3.077	7.692	0.000	63.077	1.538	63.077	0.000	7.692	0.000	4.615
Chile	423	1.182	0.000	0.236	0.000	12.530	0.236	1.182	0.000	0.236	0.000	0.000
Uruguay	93	93.548	2.151	1.075	0.000	68.817	2.151	67.742	2.151	0.000	0.000	0.000
Panamá	11	100.000	0.000	27.273	0.000	100.000	18.182	54.545	0.000	9.091	0.000	0.000

### Fuzzy-inclusion relationships between models

In Fig. 2 we show the values for F-*Ae. aegypti*, F-*Ae. albopictus* and F-dengue inclusion into one another and in Fig. 3 the values of the inclusion of the two vector-dengue intersections one into the other. The main results were that F-dengue was included in a higher proportion into F-*Ae. aegypti* (0.75) than into F-*Ae. albopictus* (0.55), and that the intersection F-*Ae. albopictus* ∩ F-dengue was more included into the F-*Ae. aegypti* ∩ F-dengue (0.99) model than *vice versa* (0.69).

### Dengue risk map in the current biogeographical context of vector-dengue interaction

**Fig. 4:**
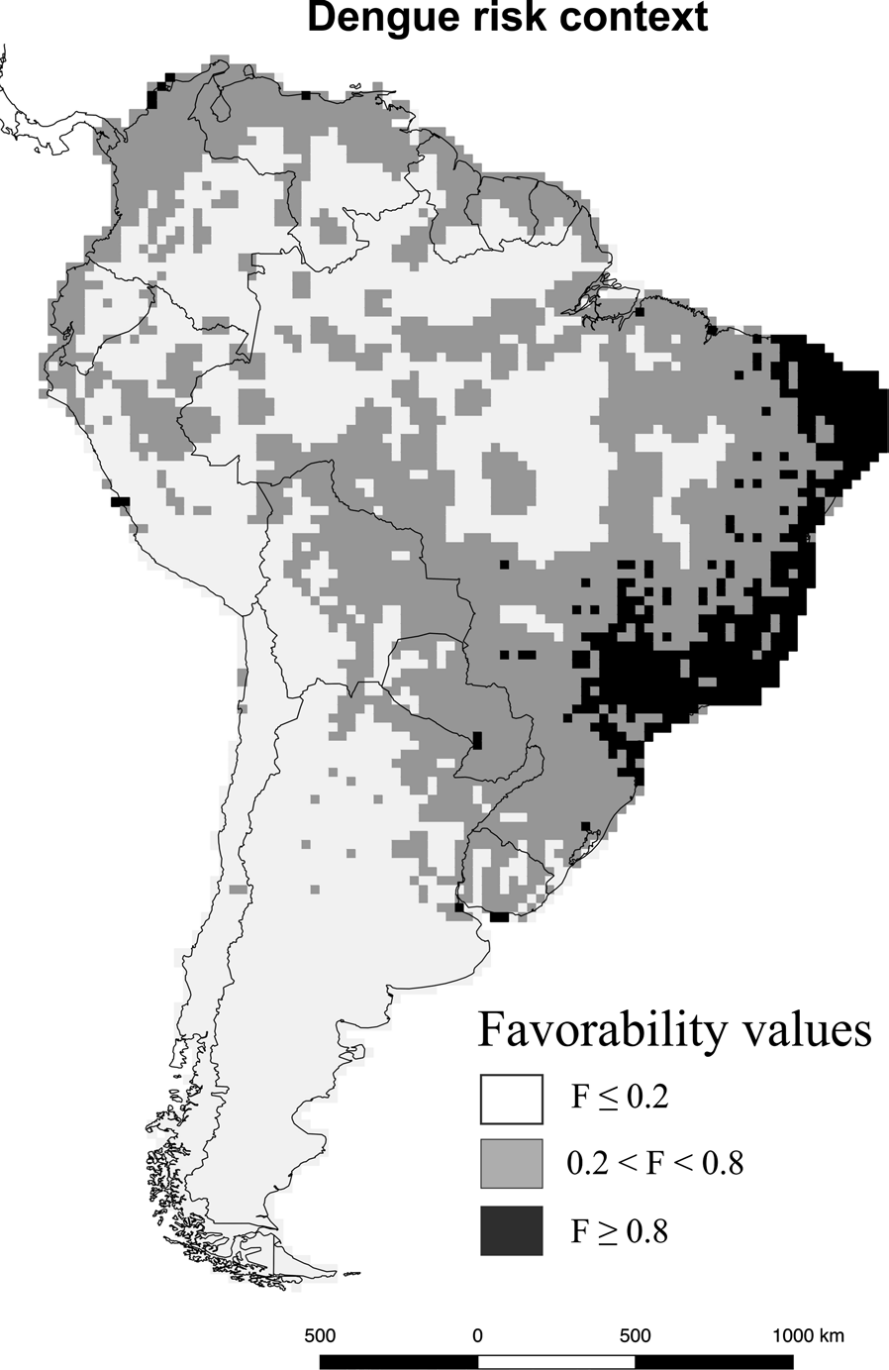
Dengue risk map in South America in the current biogeographical context of vector-dengue interaction. The map shows the intersection favorability values between the union of the favorability for the two mosquito species with the favorability for dengue (F-*Ae*. *aegypti *∪ F-*Ae. albopictus*) ∩ F-dengue. The maps were developed using QGIS (www.qgis.org) in the composer tool. The final composition was created using CorelDRAW X8

In Fig. 4, we show the comprehensive map of areas at high risk and vulnerable to dengue cases due to the two vector species combined, resulting from F-dengue ∩ (F-*Ae. aegypti* ∪ F-*Ae. albopictus*).

## Discussion

### Environmental drivers of *Aedes* vectors and dengue cases in South America

Many studies have explained the occurrence of both *Aedes* species in South America in terms of only climate [10, 25], climate and some land cover variables [7], or climate and economic information [4]. In general, they detected that temperature was the main factor limiting the distribution of the two *Aedes* species. In contrast, our favorability models detected a more complex pattern of drivers for the presence of these vectors (Table 2). Spatial structure and closeness to urban centers were among the most relevant variables for both *Aedes* species, while mean annual temperature was more important for *Ae. aegypti* and topography was more relevant for *Ae. albopictus*.

The most important drivers of dengue cases, according to our model, are the same as those favoring *Ae. aegypti*, including temperature (Table 2), which coincides with the conclusion of Capinha et al. [61]. As Campbell et al. [10] suspected, requirements for the presence of *Ae. aegypti* in South of America better reflect the risky environmental conditions for dengue occurrence than those for *Ae. albopictus*. Our results also concur with what Messina et al. [62] and Brady et al. [25] suggested, that the distribution of dengue occurrences is better modelled by incorporating drivers of different nature, such as climate, topography and human activities.

### Distribution of favourable areas

Although our explanatory models were more complex than those previously described, we detected favorable regions for both *Aedes* species coarsely similar to those described by other authors [7, 10, 25]. Areas highly favorable for *Ae. albopictus* were mostly located in Brazil and Paraguay (Fig. 2). High-favorability territories for *Ae. aegypti* were more concentrated in eastern South America, in Brazil, and some high-favorability territories reached further south than those indicated by Kraemer et al. [7], particularly in Uruguay (Fig. 2).

The dengue favorability model got lower discrimination and classification scores than the vector models (Table 3). This may result from the fact that, as other authors have pointed out [63, 64], accuracy of distribution models gets worse when a distribution is more poorly known. The published distribution data of this disease shows a scattered pattern that point to some possible bias in the quality of dengue-virus infection reports, despite the effort of Messina et al. [14]. Nevertheless, Bhatt et al. [24], by using descriptors based on climate, vegetation and human variables, described a pattern of dengue risk in South America similar to our F-dengue model (Fig. 2). However, they did not define risk areas in southern countries such as Chile and Uruguay, while we obtained areas vulnerable or at high risk in these countries. These areas represent a risk for dengue that was hidden up to now (Fig. 2). In the case of Chile, the vulnerable zones are restricted to a few low-altitude grids that were also favorable for *Ae. aegypti*. It should be noted that we found areas at high risk and vulnerable in many squares neighboring those with reported cases. This suggests that, although these areas are apparently dengue-free, they are in fact at high risk, and extreme precautions and management, control and prevention plans should be applied there.

The greatest risk for the disease in South America may be considered to occur in areas favourable to dengue (F-dengue) with reported presence of vectors *Aedes aegypti* and/or *Ae. albopictus* (Figs. 2, 4): much of Brazil and scattered regions of Venezuela, Colombia, Peru and Paraguay for *Ae. aegypti*; and much of Brazil for *Ae. albopictus*. The case of Uruguay is particularly interesting. In this country, the F-dengue model detected vulnerable locations in areas where no dengue cases had been reported for a century [11]. Uruguay was classified by Brady et al. [65] as with complete or good evidence consensus on dengue absence. However, in the summer of 2016, about 20 cases of indigenous dengue occurred in Montevideo city [11], specifically where our F-dengue model indicated a high risk of dengue occurrence (Fig. 4). Taking into account that these cases have not been considered as presences for model training in this work, this supports the predictive capacity of our model. According to Real et al. [66], the favorability function may be considered to be, for the distribution of species, analogous to what the wave function is for the distribution of quantum particles, a mathematical conceptualization of the forces that are behind the distribution of the species. This being the case, favorability values could be a better representation of the distribution of a species than the dataset of specific observations, which are always incomplete and contingent on the observation effort.

### The biogeographical context of dengue risk

In agreement with the proposal of Messina et al. [62], our approach of biogeographical modelling and fuzzy logic applied to the interaction with *Aedes* vectors has proved to be a useful method for unravelling the biogeographical context of dengue cases. The maximum simultaneous vector-dengue favorability occurs in much of Brazil for both species (Fig. 3). Additionally, some scattered areas of Colombia, Venezuela, Paraguay, Peru and Uruguay are simultaneously favorable for *Aedes aegypti* and dengue occurrence, all of them areas where disease cases have been recorded.

The F-dengue model was more included into the F-*Ae. aegypti* model than into F-*Ae. albopictus* (Fig. 2), which indicates that the favorability for *Ae. aegypti* explained to a higher extent than that for *Ae. albopictus* the dengue cases in South America. Coinciding with Campbell et al. [10], these results indicate that the distribution of *Ae. aegypti* mostly encompasses the biogeographical framework of dengue in South America, which also suggests that this species is the principal vector responsible for the dengue cases in the continent. Some authors already reported that the increase in the cases of dengue in Brazil and Argentina, for example, was directly linked to the expansion of *Ae. aegypti* [67–69]. Brathwaite et al. [13] also found a relationship between an increase in dispersion of *Ae. aegypti* between 2001 and 2010 in America and a corresponding increase in dengue virus circulation. In our analyses, compared to the model built on dengue cases alone, the model based on the intersection between dengue and *Ae. aegypti* included 26% more no-case-record locations within areas of low risk (F ≤ 0.2), i.e. had a higher specificity (Table 3). This corroborates that incorporating vector information in the biogeographical analysis of disease drivers provides a more plausible explanation about the pattern of cases occurrence [28, 29], which was previously suggested specifically for dengue as well [10, 62].

Although both mosquito species are known to act as vectors of dengue, 99% of the F-*Ae. albopictus* ∩ F-dengue model was included in the F-*Ae. aegypti* ∩ F-dengue model (Fig. 3). In addition, while the favorability for *Ae. aegypti* seems to effectively limit that for dengue (Fig. 3a), this does not happen with the favorability for *Ae. albopictus*, particularly when the favorability for the mosquito is 0.3 or lower (Fig. 3b). Consequently, in South America, in order to manage the epidemiological risk of new dengue cases, the intersection between *Ae. aegypti* and dengue favorability should be used as a the most parsimonious map of dengue risk. Nevertheless, a few territories at risk of dengue were attributed exclusively to the F-*Ae. albopictus* model. Therefore, the most appropriate risk map should include the interaction of all vectors and cases of dengue, which can be readily obtained using fuzzy logic. The fuzzy intersection between the favorability for dengue cases and the fuzzy union of the favorability for any of the vector species provided a comprehensive map of the biogeographical risk for dengue (Fig. 4). This proposal of a risk map for dengue in South America is based on the geographical-environmental, disease-trait and human profiling that constitute the starting point for risk assessments in pathogeography [29].

## Conclusions

Our results corroborate that incorporating vector information in the biogeographical analysis of disease drivers provides a more plausible explanation about the pattern of cases occurrence, and confirm that fuzzy logic is an appropriate conceptual and operational tool to deal with the nuances of the vector-illness biogeographical interactions. Thus, the application of fuzzy logic may help health authorities to better prevent, control and mitigate vector-borne diseases.

## Supplementary information

**Additional file 1: Table S1.** Dengue cases, *Aedes aegypti* and *Ae. albopictus* occurrences (presences as 1, and absences as 0 in each South America grid), values of the significant variables in the models by South America grid, and favorability values for each model by grid (F-dengue; F-*Ae*. *aegypti* and F-*Ae. albopictus*). Variable abbreviations are given in Table 1.

## Data Availability

We have included the data table used as Additional file 1: Table S1.

## Abbreviations

SDM: species distribution models
AIC: Akaike’s information criterion
F: environmental favorability
AUC: area under the curve
CCR: correct classification rate
